# Pan-Cancer Analysis of Clinical Relevance via Telomere Maintenance Mechanism

**DOI:** 10.3390/ijms222011101

**Published:** 2021-10-14

**Authors:** Ji-Yong Sung, Jae-Ho Cheong

**Affiliations:** 1 Department of Biomedical Systems Informatics, Yonsei University College of Medicine, Seoul 03722, Korea; jiyongsung@yuhs.ac; 2 Department of Laboratory Medicine, Yonsei University College of Medicine, Seoul 03722, Korea; 3 Department of Surgery, Yonsei University College of Medicine, Seoul 03722, Korea; 4 Yonsei Biomedical Research Institute, Yonsei University College of Medicine, Seoul 03722, Korea; 5 Department of Biochemistry & Molecular Biology, Yonsei University College of Medicine, Seoul 03722, Korea; 6 Department of Research & Development, VeraVerse Inc., Seoul 03722, Korea

**Keywords:** telomere maintenance mechanism, non-defined telomere maintenance mechanism, alternative lengthening of telomere

## Abstract

Understanding the telomere maintenance mechanism (TMM) in immortal cancer cells is vital for TMM-targeted therapies in clinical settings. In this study, we classified four telomere maintenance mechanisms into telomerase, ALT, telomerase + ALT, and non-defined telomere maintenance mechanism (NDTMM) across 31 cancer types using 10,704 transcriptomic datasets from The Cancer Genome Atlas. Our results demonstrated that approximately 50% of the total cohort displayed ALT activity with high telomerase activity in most cancer types. We confirmed significant patient prognoses according to distinct TMMs in six cancer types: adrenocortical carcinoma (ACC), PAAD, HNSC, SARC, GBM, and metastatic cancer. Patients with metastasis had a poor prognosis in the ALT group (*p <* 0.006) subjected to RAS protein signal transduction. Glioblastoma patients had poor prognosis in NDTMM (*p* < 0.0043) and showed high levels of myeloid leukocyte activation. Pancreatic adenocarcinoma (*p* < 0.04) and head and neck squamous cell carcinoma (*p* < 0.046) patients had a good prognosis in the ALT group with high immune cell activation. Furthermore, we showed that master transcriptional regulators might affect the selection of the TMM pathway and explained why different telomere maintenance mechanisms exist. Furthermore, they can be used to segregate patients and predict responders to different TMM-targeted therapeutics.

## 1. Introduction

The telomere maintenance (TMM) mechanism is used by cancer cells to promote immortality [1]. Recently, as the research on telomerase [2] and alternative lengthening of telomeres [3] in human cancer is being actively conducted, interest in the role of TMM in the immortality of tumor cells (which is one of the hallmarks of cancer) is increasing. It has also been studied in cancer cell lines with tumors of relevant origin based on TERT isoform expression patterns [4].

In most cancer cells, telomerase activity is maintained; however, some cancer cells, such as telomerase-deficient cancer cells, use an alternative lengthening of the telomeres (ALT) mechanism for their survival [5]. Telomere lengthening is mainly mediated by TERT (telomerase) and ALT (ATRX/DAXX alteration); however, in approximately 22% of the samples, a non-defined telomere maintenance mechanism (NDTMM) might be involved [6]. Little is known about the NDTMM, but it has been reported in several cancer types, including glioblastoma [7], osteosarcoma [8], and metastases of cutaneous melanoma [9]. In addition, the role of telomere homeostasis in metastatic cancer is unknown, and targeting TMM in aggressive metastatic tumors with a poor prognosis can be a good strategy. Therefore, it is crucial to understand the molecular mechanisms underlying the four types of telomere maintenance mechanisms and their impact on the survival of patients. 

To study the clinical relevance of the four TMM-associated pathways, we performed a thorough assessment of their relationship with clinical prognostic indicators in various cancer types. We have comprehensively analyzed ALT activities across 31 cancer types in The Cancer Genome Atlas (TCGA) [10]. In this study, we primarily focused on the distinct molecular features related to the four TMM types and assessed their clinical relevance. The following results were obtained: First, cancer types differ significantly in prognosis according to the four TMM types. Second, various cancer types have different molecular profiles depending on the type of TMM; TMM types in pan-cancer are associated with genomic alternations [11]. Third, certain TMMs are only associated with different biological processes. The functional diversity of telomerase indicates important differences between these two TMM pathways (telomerase and ALT), which may prove to be essential in cancer for the acquisition of metastatic phenotypes [12].

Therefore, our goals were to refine our understanding of the four TMM types and use this framework to identify drug targets that can be harnessed to overcome TMM-type-based resistance.

## 2. Results

### 2.1. Telomere Maintenance Mechanism Separated Patient Outcome

**Figure 1:**
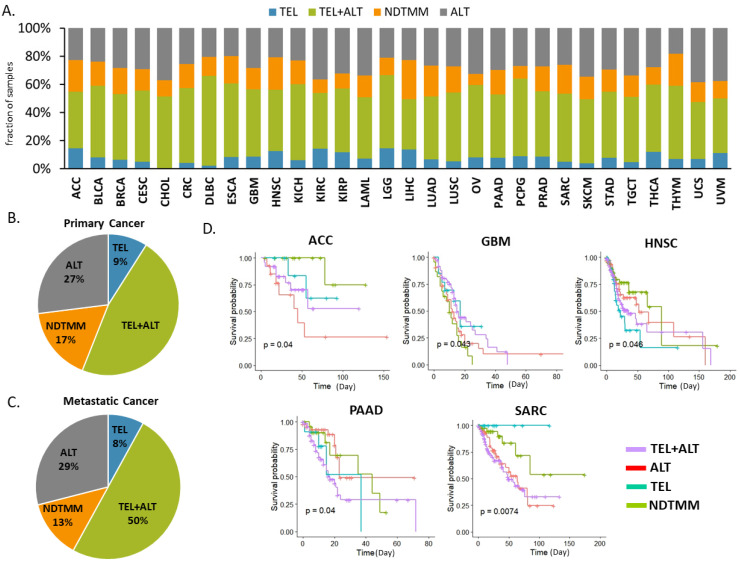
Telomere maintenance mechanism and survival probability for 5 cancer types. (**A**) The percentage of the four types of telomere maintenance mechanisms in tumor samples; TEL: telomerase, TEL+ALT: telomerase and alternative lengthening of telomere, NDTMM: non defined telomere maintenance mechanism, ALT: alternative lengthening of telomere. (**B**) Pie chart showing the frequency of four telomere maintenance mechanisms in primary tumors of 31 cancer types, TEL: 9%, TEL+ALT: 47%, NDTMM: 17%, and ALT: 27%. (**C**) Pie chart showing the frequency of the four telomere maintenance mechanisms in metastatic tumors of 11 cancer types, TEL: 8%, TEL+ALT: 50%, NDTMM: 13%, and ALT: 29%. (**D**) Kaplan–Meier plots showing the overall survival rates of patients classified according to the four telomere maintenance mechanisms. The *p*-value was calculated using the log-rank test. Five cancer types (ACC, GBM, HNSC, PAAD, and SARC) had significantly different prognoses. ACC, adrenocortical carcinoma; GBM, glioblastoma multiforme; HNSC, head and neck squamous cell carcinoma; PAAD, pancreatic adenocarcinoma; and SARC, sarcoma.

**Figure 2:**
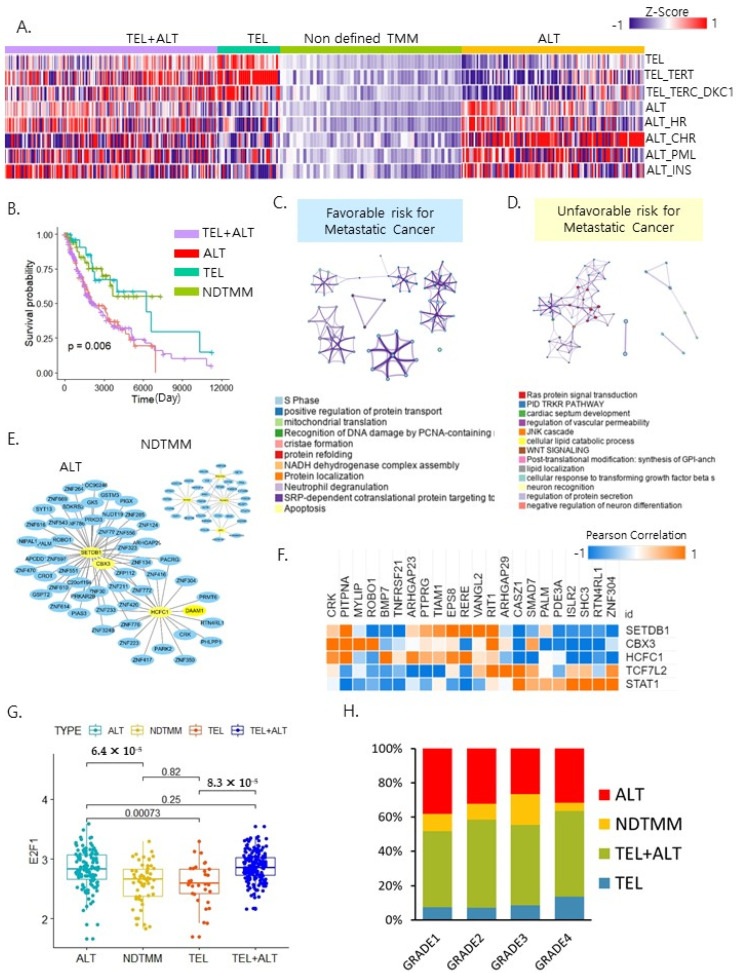
Telomere maintenance mechanism in metastasis cancer. (**A**) Heat map of four types of telomere maintenance mechanisms in metastasis cancer. TEL+ALT: telomerase and alternative lengthening of telomere, TEL: telomerase, NDTMM: non–defined TMM (no/low activity of telomere maintenance mechanism), ALT: alternative lengthening of telomere. (**B**) Kaplan–Meier plots showing the overall survival rates for the four types of TMM in metastasis cancers. (**C**) Enriched biological process in ALT samples. (**D**) Enriched biological process in NDTMM samples. (**E**) Transcriptional factors and their target genes for ALT in metastasis cancer. (**F**) Heat map of correlation between S phase and significant transcriptional factors (orange: positive, blue: negative). (**G**) Box plot of *E2F1* expression in the four TMM types. (**H**) Frequency of TMM for each tumor grade.

To classify the telomere maintenance mechanisms, we used TCGA RNA-sequencing data from 31 cancer types with pooled metastatic tumor samples from 11 cancer types (Figure 1A, Supplementary Data Table S1). Four telomere maintenance mechanisms were defined [10] according to the TMM signature [13]. To classify the TMM subtype using transcriptome data, we used a single-sample gene enrichment score for a single patient sample (ssGSVA) [14]. Then, we split the samples into four types: telomerase, telomerase + ALT, ALT, and NDTMM samples for each cancer type (Figure 1A). Among 10,704 samples, 47% displayed both telomerase and ALT, 27% displayed ALT, 9% telomerase, and 17% NDTMM [6] (Figure 1B). The four TMM activities varied across the cancer types. Cholangiocarcinoma (CHOL) showed no telomerase activity among the 31 cancer types. We also calculated the telomere maintenance mechanism in metastatic tumor samples from the TCGA, with 11 cancer types. The frequency of telomere maintenance mechanism types in metastatic tumors was similar to that of primary tumor samples (Figure 2C). In five cancer types, namely, adrenocortical carcinoma (ACC), pancreatic adenocarcinoma (PAAD), head and neck squamous cell carcinoma (HNSC), sarcoma (SARC), and glioblastoma multiforme (GBM), the four types of telomere maintenance mechanism presented significant prognostic value (SARC: *p* = 7.4 × 10^−3^, ACC: *p* = 4.0 × 10^−2^, GBM: *p* = 4.5 × 10^−2^, PAAD: *p* = 4.0 × 10^−2^, HNSC: *p* = 4.6× 10^−2^) (Figure 1D). GBM had a poor survival rate for NDTMM [15], and the ALT groups of GBM showed poor survival rate. Although NDTMM has only been reported in certain cancer types [1], our results showed that NDTMM could function in all cancer types. 

In contrast, ACC with NDTMM had a good survival rate. ACC and GBM with ALT displayed the opposite trend regarding survival rate. Two cancer types with the telomerase mechanism, PAAD and HNSC, had poor outcomes. In addition, high ALT levels (*p* = 0.04) were associated with a better prognosis of PAAD. Overall, our analyses showed that TMM type might distinguish patient prognosis in a single patient sample and can be used as a prognostic marker.

### 2.2. ALT Was Associated with a Poor Prognosis of Metastatic Cancer

Sarcoma (SARC) with high ALT was shown to have poorer prognoses in comparison with low ALT [10]. However, there are few studies on TMMs in metastatic cancer. We hypothesized that the selection of TMMs in cancer cells is a critical factor for their metastatic potential. Metastatic tumors had TMM types similar to primary tumors (Figure 1C and Figure 2A). Approximately 29% of the metastatic samples displayed ALT activation (Figure 1C). We compared the survival rate of patients in the four TMM groups from metastatic tumor samples, including 11 cancer types. As expected, ALT was associated with a poor prognosis, and NDTMM with a good prognosis (*p* < 0.006) (Figure 2B). Next, we performed gene ontology analysis to obtain functional insights into the clinical outcomes of NDTMM and ALT. The poor outcome-related biological pathway for ALT was “ras protein signal transduction,” and the good outcome-related biological pathways for NDTMM were “S phase” and “mitochondrial translation” (FDR = 0.001) (Figure 2C,D). These results show that ALT in metastatic tumors is associated with RAS signaling and Wnt signaling. One of the most obvious hallmarks of cancer is uncontrolled proliferation of cells, partly due to its independence from the growth factor supply. Ras, a small GTPase, is a major component of mitogenic signaling [16]. WNT signaling and metastasis are particularly associated because WNT/β-catenin signaling can regulate the expression of multiple telomeric proteins [17]. 

Next, analysis of transcriptional factor (TFs), as master regulators, indicated that the favorable risk with NDTMM may be regulated by the transcriptional factors *NFKB1*, *RUNX3*, *SPI1*, and *POLR2A* (FDR = 0.0001) (Figure 2E), whereas the unfavorable risk with ALT may be regulated by *SETDB1*, *CBX3*, *HCFC1*, *TCF7L2*, and *STAT1* (FDR = 0.0001) in the “Ras protein signal transduction” pathway, including the gene *RIT1* in “RET signaling” pathway (Figure 2F). Expression of E2F1 (Figure 2G), as an hTERT repressor TF, was significantly different between ALT (*p* = 6.4 × 10^−5^) and NDTMM. We then assessed the frequency of pathologic tumor stage in the four distinct TMM groups. Interestingly, NDTMM was highly associated with grade 3, but NDTMM was present in a small fraction of grade 4 (Figure 2H). These results demonstrate that TMMs may differentially contribute to tumor progression of metastatic cancer. Overall, TMM types in metastatic cancer have a frequency similar to that in primary tumors, but master regulators and signaling pathways in ALT were different from those reported in a previous study. Therefore, the type of TMM may be a useful prognostic marker in patients with metastatic cancer.

### 2.3. Molecular Characteristics Based on the Four TMM Types 

*ATRX* and *DAXX* gene mutations might be more generally associated with the ALT phenotype [18], and *TERT* promoter mutations enhance telomerase activation [19]. We focused on six cancer types with distinct prognoses and identified their molecular characteristics in each specific TMM type.

Repair of dysfunctional telomeres by fusion propels cells into breakage–fusion–bridge cycles, resulting in unequal distribution of genetic material into daughter cells, and, hence, genome instability [20]. Telomere dysfunction increases mutation rates and genomic instability [21]. Next, we analyzed the copy number variation and tumor mutation burden profiles of 1201 primary cancer specimens and 395 metastatic cancer specimens across six cancer types with pooled metastatic tumor samples.

**Figure 3:**
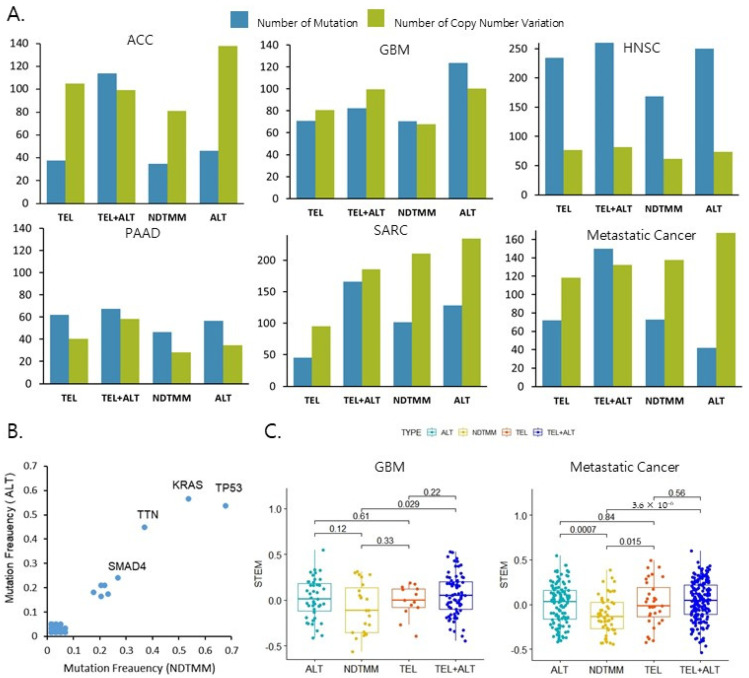
Molecular characteristics of five primary cancer types and metastatic cancers. (**A**) Bar chart depicting the number of mutations and copy number variation that were identified per cancer type (ACC, GBM, HNSC, PAAD, SARC, and metastatic cancer). (**B**) Scatter plot showing the mutation frequency between ALT and NDTMM. (**C**) Boxplots showing differences in stemness enrichment score levels among the four TMM types for GBM and metastatic cancer.

ACC and SARC showed significantly higher copy number variations in the ALT group than in the NDTMM group (Figure 3A). Interestingly, several cancer types, including HNSC and PAAD, displayed a high tumor mutation burden (TMB) in the four TMM types. Metastasis cancer showed a similar pattern to ACC in four TMM types. For ACC and metastatic cancer, the patient prognosis according to TMMs was the same. We found that high copy number variation (CNV) in ALT was associated with poor prognosis for ACC, SARC, and metastatic cancer (Figure 2B). The five cancer types showed significantly different mutation frequencies between ALT and NDTMM. *KRAS* was the most frequently mutated gene in ALT and *TP53* was frequently mutated in NDTMM for five cancer types (Figure 3B). We confirmed a significant difference in stemness (*p* < 0.0007) between ALT and NDTMM in metastatic cancer. Both telomerase and ALT activity may cause high stemness in GBM (Figure 3C). This result suggests that in six types of cancers a specific telomere maintenance mechanism is associated with genomic instability of copy number variation and mutation during cellular proliferation. In particular, in the case of ALT, it was confirmed that the prognosis was poor compared to other TMM types; ALT type was associated with relatively high copy number variation, and this result may provide a critical clue to the synthesis of non-canonical telomeric DNA. Together, these studies indicate that subtelomeres are hotspots of DNA breakage and repair, and are likely to be responsible for the generation of complex interchromosomal duplication patterns and the rapid evolution of these genomic regions, as well as the prevalence of large CNVs near telomeres [22]. 

### 2.4. Different Biological Processes Affected Patient Prognoses of Different TMM Groups

**Figure 4:**
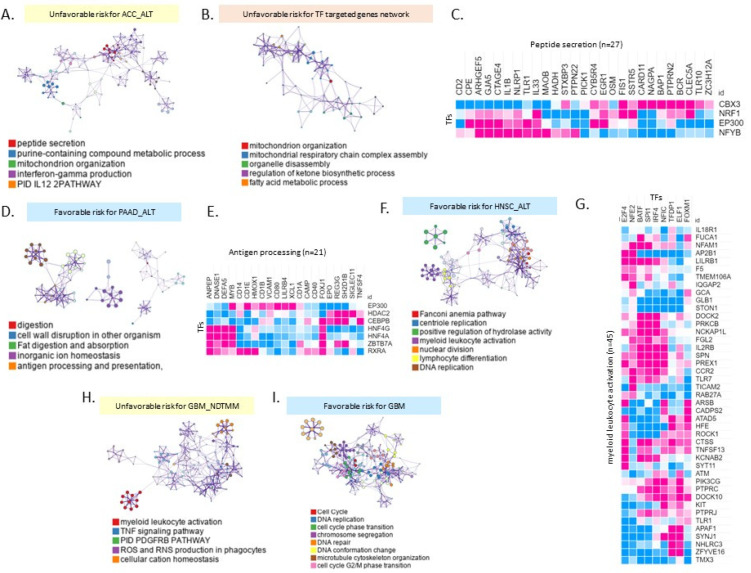
Visualized enrichment networks of gene ontology analysis of good/poor prognosis specific mRNA expression. (**A**) Gene ontology analysis of unfavorable risk for the differentially expressed genes (DEGs) between the good patient outcome group and poor patient outcome group in ACC with ALT. (**B**) TF targeted gene network in unfavorable risk ACC with ALT. Top 5 enriched pathways showing significant difference (FDR = 0.001). (**C**) Heatmap of correlation value between peptide secretion signature genes (n = 27) and transcriptional factors (CBX3, NRF1, EP300, and NFYB) (pink: positive correlation, blue: negative correlation). (**D**) Gene ontology analysis of favorable risk of PAAD with ALT. (**E**) Heatmap of correlation matrix between antigen processing (n = 21) and transcriptional factors (EP300, HDAC2, CEBPB, HIF4G, HIF4A, ZBTB7A, and RXRA). (**F**) Gene ontology analysis of favorable risk of HNSC with ALT. (**G**) Heatmap of correlation matrix between myeloid leukocyte activation (n = 45) and transcriptional factors (E2F4, NFE2, BATF, SPI1, NFIC, TFDP1, ELF1, and FOXM1). (**H**) Gene ontology analysis of unfavorable risk of GBM with NDTMM. (**I**) Gene ontology analysis of favorable risk GBM with ALT.

We confirmed different patient prognoses according to the four TMM types (Figure 1D). We performed a gene ontology analysis to obtain functional insights into TMM types according to clinical outcomes. Poor outcome-related biological pathways enriched in ACC with ALT were related to peptide secretion, purine-containing compound metabolic process, mitochondrion organization, and interferon-gamma production (Figure 4A).

Next, we analyzed the transcription factors and target gene networks. The mitochondrion organization, mitochondrial respiratory chain complex assembly, organelle disassembly, and regulation of ketone biosynthetic processes (FDR = 0.001) were enriched in ACC with ALT (Figure 4B). Mitochondrial biogenesis was higher in the ALT group than in the telomerase group according to a previous study [23]. The vulnerability of the mitochondrial genome to mutations and the somatic mutations promote poor prognosis [24]. 

The unfavorable risk of ACC with ALT may be determined by the transcriptional factors *CBX3*, *NRF1*, *EP300*, and *NFYB* (FDR = 0.001) (Figure 4C). PAAD and HNSC with ALT and favorable risk were enriched in immune-related biological pathways such as antigen processing and myeloid leukocyte activation (FDR = 0.001) (Figure 4D,F). *EP300*, *HDAC2*, *CEBPB*, *HNF4G*, *HNF4A*, *ZBTB7A*, and *RXRA* genes were correlated with antigen processing-related genes for favorable risk of PAAD with ALT (Figure 4E). Myeloid leukocyte activation was regulated by *E2F4*, *NFE2*, *BATF*, *SPI1*, *IRF4*, *NFIC*, *TFDP1*, *ELF1*, and *FOXM1* genes in HNSC with ALT (Figure 4G). In GBM, the poor outcome related to NDTMM and was enriched in myeloid leukocyte activation, TNF signaling pathway, PDGFRB pathway, ROS, and RNS production in phagocytes (Figure 4H). Favorable risk for GBM is related ALT and enriched in cell cycle, DNA replication (Figure 4I).

Overall, our analyses showed that different biological processes might affect the four TMM types in an individual sample of a specific cancer type. 

## 3. Discussion

The telomere maintenance mechanisms play essential role in the immortalization of cancer cells, and tumor cell survival is mainly maintained by two mechanisms: telomerase and alternative lengthening of telomeres. In a previous study, 22% of all TCGA cancers did not express TERT or had mutations in ATRX or DAXX [6]. The frequency of ALT occurrence varies by cancer types. A higher rate of ALT activation was reported in tumors of mesenchymal origin than in carcinomas of epithelial origin. However, the reason for this is still not clearly known [25].

Although it is known that ALT occurs frequently in sarcoma and brain tumors, ALT also occurs not infrequently in several epithelial cancer types (adrenocortical carcinoma: 12% [26], ganglioneuroblastoma: 14% [27], neuroblastoma: 34% [28], osteosarcoma: 64% [8], synovial sarcoma: 9% [29], breast cancer: 2% [30], astrocytoma:42% [31], glioblastoma: 28% [32], colorectal cancer: 6% [33], kidney cancer: 5% [27], liver cancer: 7% [34], lung cancer: 1% [35], carcinoid tumor: 6% [27], PanNET: 53% [36], paraganglioma: 13% [27], ovary cancer: 1% [27], melanoma: 7% [37], soft tissue of malignant fibrous histiocytoma: 62% [38], leiomyosarcoma: 58% [39], liposarcoma: 25% [40], gastric carcinoma: 19% [41], MSI-H gastric carcinoma: 57% [41], non-MSI-H gastric carcinoma: 19% [41], testis cancer: 8% [27], medullary thyroid carcinoma: 28% [42], urinary bladder: 4% [27], uterus: 2% [27]).

It has been reported that about 19% of ALT gastric cancers occur in tumors with MSI high, but it has been recently reported that about 30% of gastric cancer occur ALT in the stem-like molecular type [43], suggesting that ALT frequency may depend on the molecular subtypes. Pertinent to this, since the frequency of ALT activity may be different for each molecular subtype in individual cancer types, there would be discrepancy between observed and predicted ALT frequency according to the composition of subgroups in population of evaluation. Thus, it might explain a difference between the previously reported frequency of ALT activity and that predicted in this study.

Telomerase and ALT in some cancer types (glioblastoma multiforme [7], osteosarcomas [8], soft tissue sarcomas [44], liposarcomas [45], fibrous histyocytomas [38], peritoneal mesothelioma [46], adrenocortical carcinoma [26], gastric carcinomas [41]) may coexist [47].

In this study, we showed that NDTMM occurs in 30 cancer types (96.77%). In ACC, SARC, and metastatic cancer, samples with NDTMM had the best prognosis, but the prognosis was poor in the ALT group. We confirmed that ALT in metastatic cancer is related to the RAS protein signaling pathway.

This suggested that ALT could use different signaling pathways for each cancer type.

In the ALT groups of ACC, SARC, and metastatic cancer, poor outcome-related molecular profiles were associated with significantly higher CNV. PAAD and HNSC showed relatively good prognosis in the telomerase group, and high immune cell activation, such as antigen-presenting cells and myeloid leukocyte activation, was confirmed in the ALT group. This is the first study to show that two cancer types, PAAD and HNSC, have a better prognosis in the telomerase group than in the ALT group. In addition, we confirmed that higher TMM activation was associated with higher stemness in metastatic cancer. Alternative lengthening of telomeres is important for epidermal homeostasis and tumorigenesis in cancer stem cells [48]. Although our study is limited to bioinformatics analysis and has a limited number of samples depending on the type of TMM, future study is needed to assess associated candidate pathway genes for six cancers associated with TMM types.

## 4. Materials and Methods

### 4.1. Telomere Maintenance Mechanism Classification

To test the telomere maintenance mechanism, we used single-sample gene variation analysis (ssGSVA) [14] of 31 RNA-seq data from TCGA. The TCGA mRNA expression dataset was obtained from Broad GDAC Firehose (https://gdac.broadinstitute.org/, accessed on 1 August 2020). The gene set used to evaluate TMM was the same as that used in a previous study [13]. We performed 100,000 or more runs to increase the statistical significance. We classified four types of TMM per cancer, and the criteria for classification was to find the TMM with the highest relative activity among the four types and identify the sample. TEL, relatively high telomerase activity; ALT, ALT activity; NDTMM, non-defined telomere maintenance mechanism with no or low telomerase activity; and TEL+ALT, ALT activity with telomerase activity.

### 4.2. Differential Expression Gene Analysis in Cancer Types

We performed DEG analysis for the good outcome samples compared to the poor outcome samples, as well as the samples with NDTMM compared to the samples with ALT in six cancer types (ACC, GBM, HNSC, PAAD, SARC, and metastatic cancer) using the “Limma” R package [49].

### 4.3. Survival Probability Analysis and Gene Ontology and Correlation Analysis

The R package ‘‘survival’’ [50] was used to perform the overall survival analysis and produce the Kaplan–Meier survival plots. A log-rank test was used to assess the statistical significance (*p <* 0.05). Gene ontology analysis was performed using METASCAPE [51] and DEGs (FDR < 0.05).

### 4.4. Transcription Factor Analysis Protein Association Network

We identified transcription factors (TFs) and target genes using the Cytoscape plug-in iRegulon, which pairs motifs and chromatin immunoprecipitation-sequencing (ChIP-seq) tracks to determine the TFs controlling gene networks, and the iRegulon database (version 2015.02.12) [52]. We focused on six main TMM pathways and signature gene sets [13].

## Data Availability

Not applicable.

## Supplementary Materials

All data are available online at https://www.mdpi.com/article/10.3390/ijms222011101/s1.
